# Subliminal Semantic Processing of Grasping Actions: Evidence from ERP Measures of Action-Verb Priming

**DOI:** 10.3390/bs16020206

**Published:** 2026-01-30

**Authors:** Yanglan Yu, Anmin Li

**Affiliations:** School of Psychology, Shanghai University of Sport, Shanghai 200438, China; 15295760038@163.com

**Keywords:** grasping manipulative actions, embodied semantic recognition, subliminal visual consciousness, event-related potentials (ERPs)

## Abstract

Human interaction with manipulable objects relies heavily on the ability to perceive and execute grasping actions, yet it remains unclear whether the semantics of these actions are processed without conscious awareness. While previous work has identified bottom-up influences on grasp recognition, direct evidence for subliminal semantic processing of grasping actions is limited. Grounded in embodied cognition theory—which posits overlapping neural mechanisms for action language and action execution—the present study examined whether grasp-related verbs can elicit subliminal priming effects on grasping-action recognition. Using a masked priming paradigm, participants classified objects requiring either precision or power grasps while subliminal Chinese action verbs served as primes. Behavioral measures revealed faster responses for semantically congruent cue–target pairs. ERP analyses further demonstrated congruency effects in the N400 and P600 components, reflecting semantic integration and conflict monitoring, as well as modulation of the P300 associated with action-related evaluation. Both grasp types showed evidence of unconscious semantic processing, though precision- and power-grasping actions produced distinct neural patterns. These findings provide direct experimental support for subthreshold semantic activation of grasping actions and confirm the viewpoint of action-language-embodied processing. The study advances the theoretical understanding of unconscious-action semantics and offers a framework for investigating how manipulative-action meaning is accessed below the threshold of awareness.

## 1. Introduction

Human societies are fundamentally shaped by tools, and our pervasive use of manipulable objects has long motivated research on the cognitive- and motor processes supporting their operation (Gibson & Ingold, 1994). Manipulable objects occupy a unique dimension in which actions and objects form tightly coupled representations: through manipulation, objects change shape or achieve specific functional goals (Preston, 2012). Objects that afford such functionality are termed manipulable objects (Brandi et al., 2014), and the human repertoire for interacting with them primarily includes grasping actions, which involve tactile acquisition and lifting, and using actions, which apply tools toward functional ends (Brandi et al., 2014; Errante et al., 2021; Fagg & Arbib, 1998).

The ability to reach for and manipulate objects emerges early in development—typically by age four (Michel & Claude, 1999)—and relies on complex perceptual–motor integration (Miall et al., 2019). When viewing a manipulable object, the human brain rapidly extracts the appropriate grasp: a power grasp for a hammer or a precision grasp for a pencil (Bergstrom et al., 2021). Research suggests that this mapping remains stable across perspectives and visual contexts. Central to this process is the formation of structural action representations, which encode an object’s current size, shape, and orientation to guide goal-directed grasping (Moguilner et al., 2021). Because these representations depend on immediate perceptual input, they place minimal strain on working memory but remain active for a brief temporal window (Buxbaum & Kalenine, 2010).

A key question in cognitive neuroscience concerns the boundary between conscious and unconscious action recognition. Some studies propose that grasping-action processing can proceed independently of selective visual attention (Fu et al., 2018). For example, enhanced neural potentials emerge when participants observe typical action–object pairings (hand–pencil) even when attention is diverted (Senna et al., 2014). Subliminal stimuli can also induce response-hand congruency effects driven by handle orientation (Pappas & Mack, 2008), and masked object images can prime categorical decisions (Almeida et al., 2008). Additionally, unconscious recognition processing relatively supports grasping action when observing objects’ manipulable features (Fang & He, 2005). However, such priming may sometimes reflect sensitivity to simple shape features—such as elongated objects—rather than true grasp semantics (Ludwig et al., 2015). Thus, direct evidence of subliminal semantic processing for grasping actions remains limited.

Embodied cognition provides a strong theoretical motivation for addressing this gap. Action language—especially verbs with strong manipulability features—engages overlapping neural circuits with action execution (Beauprez et al., 2020; Madan, 2014). Action-related words facilitate recognition of visually presented actions and manipulable objects (Arevalo et al., 2007; Liu et al., 2022; Monaco et al., 2023). Verb cues in particular yield stronger and longer-lasting priming effects than nouns or images (Ball et al., 2025), and grasp-related verbs reliably evoke P300 components (Lee et al., 2018). Manipulability further modulates action-language processing, as hand- and foot-related vocabulary activates corresponding sensorimotor regions (Klepp et al., 2014).

In Chinese, action verbs possess unique linguistic properties: they often carry related semantic meaning even in isolation, without contextual support (Deng et al., 2016). That is to say, transitivity, as a grammatical feature, is not affected by the completeness of sentence structure and can effectively indicate semantics. For example, single-word Chinese verbs can still activate a significant N400 component in the absence of an object, supporting the view that grammatical features do not affect semantics. This characteristic, combined with the strong action–language coupling described by embodied theories, raises a central question: Can the semantic content of grasping actions be processed at a subliminal level? And more specifically: Does masked verb processing modulate the recognition of objects requiring different grasp types?

To address these questions, the present study focused on two grasping categories—precision- and power grasps—which differ both in motoric configuration and in Chinese verb semantics. Chinese action verbs exhibit a stable transitivity feature, which is inherent to their grammatical structure and does not depend on the completeness of the surrounding context. Although the characters representing precision grasps and power grasps may share similar radicals, their meanings are distinct, illustrating that single-character verbs in Chinese can convey different semantic and syntactic information (Deng et al., 2016).

Using electroencephalography (EEG), we analyzed the temporal dynamics of ERP components associated with visual processing, semantic conflict (Leynes et al., 2024; Li & Wang, 2016), manipulative action recognition (Lee et al., 2018), and late semantic reanalysis (Emmorey et al., 2025; Kim et al., 2024). Our investigation aimed to provide direct evidence for subliminal semantic processing of grasping actions and to clarify how different grasp types shape unconscious semantic activation. Building on previous research on embodied action-language processing, the present study further investigates whether grasp-related verbs can elicit subliminal semantic activation. We hypothesize that subliminally presented verb cues will influence participants’ behavioral responses and ERP components depending on semantic congruency. This approach provides novel linguistic evidence for subthreshold processing of grasping actions (Fang & He, 2005) and uses grasping actions as stimuli to test theories of embodied action-language processing (Courson & Tremblay, 2020; Giacobbe et al., 2022).

## 2. Materials and Methods

### 2.1. Participants

Thirty students from the Shanghai University of Sport participated in this experiment (14 males and 16 females, aged 19–24 years, mean ± SD = 20.46 ± 1.97 years). Mean body mass index (BMI) was 22.20 ± 5.16 kg/m^2^ (range, 17.36–37.86). All had normal or corrected vision, no significant differences in BMI, were right-handed, were healthy, were free from neurological or muscular diseases, and had not recently taken psychoactive medications. The experimental requirements and procedures were explained beforehand, and written informed consent was obtained. The participants were compensated on the basis of their participation time and were paid for their participation. This sample size meets the requirements of a power analysis previously calculated through G*Power version 3.1.9.2 (Faul et al., 2007), the effect size of the two factor repeated measures ANOVA is 0.25, with an alpha level of 0.05 and a power of 0.8 set for the sample-size calculation. The study followed ethical guidelines set forth by the Declaration of Helsinki and was approved by the local ethics committee.

### 2.2. Stimuli

Target images were selected from the Bank of Standardized Stimuli (BOSS) (Brodeur et al., 2014). Following prior classifications (Buxbaum et al., 2006), manipulable objects were grouped into two grasping types: precision and power. The Chinese verbs “捏” (precision grasp) and “握” (power grasp) provided distinct semantic cues without requiring context for interpretation (Deng et al., 2016).

**Figure 1:**
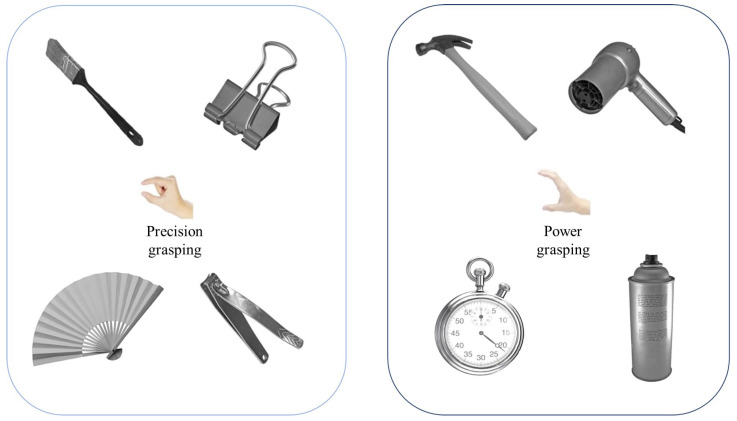
Manipulable objects Stimuli.

To validate grasp-type categorization, 198 independent participants (ages 18–25) classified the selected objects. A chi-square test confirmed strong agreement across categories (Table 1). Eight objects (four per grasp-type) were used in the experiment (Figure 1). Stimuli were grayscale, presented at a 45° left-tilted handle orientation, and subtended a visual angle of 3.8°. Images were displayed using Psychtoolbox in MATLAB 2020b version (Brainard, 1997; Pelli, 1997) on a calibrated 1024 × 768 display (60 Hz) at 45 cm. Responses were collected via keyboard, and ERP analyses examined the time course of neural activation.

### 2.3. Task and Procedure

This study is divided into two processes, the action-type testing section and the formal experimental section. The action-type testing section aims to test the participants’ selection of two types of grasping action for the target stimulus object, in order to confirm whether the participants’ choice of action type is applicable to the use of semantic priming stimulation conditions during formal testing. The action-type testing section lasts about 20 min, and the formal experimental section lasts about 60 min.

To ensure that the duration of subliminal prime presentation remained consistently below each participant’s visual awareness threshold on every trial, we first measured individual perceptual thresholds. Using a staircase procedure ranging from 17 ms to 66 ms, we determined the minimum exposure duration at which each participant could reliably detect the stimuli. The average awareness threshold across participants was 39 ms. Based on this value, we set the prime duration to 33 ms to guarantee that the stimuli were presented under subliminal conditions.

In this study, both subjective and objective measures of visual awareness were employed. During the main experiment, subjective awareness was assessed on a trial-by-trial basis. After completing the response to each target stimulus, participants verbally reported their perceptual experience of the preceding prime word using a four-point Perceptual Awareness Scale (PAS), where 1 indicated no experience at all, 2 indicated a brief glimpse, 3 indicated an almost clear experience, and 4 indicated a clear experience.

Only trials in which participants reported PAS = 1 (i.e., no subjective awareness of the prime) were classified as unconscious trials and included in subsequent behavioral and ERP analyses. Trials with PAS ratings greater than 1 were excluded from all analyses.

**Figure 2:**
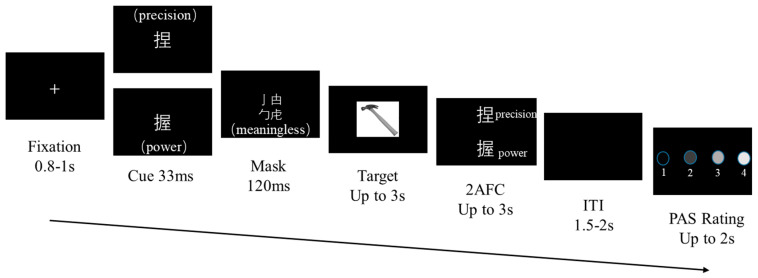
Procedure design for the main experimental phase.

The experimental procedure, informed by prior masked-priming research and refined following (Almeida et al., 2008), used a masking priming paradigm to examine how cue–target semantic congruency influences action-type judgments. Each trial began with a central white fixation cross presented for a randomized duration between 0.8 and 1 s to reduce anticipatory effects (Figure 2). A cue verb—either “捏” (precision grasp) or “握” (power grasp)—was then displayed for 33 ms, followed by a 120 ms pattern mask composed of Chinese radicals without lexical or semantic meaning. Subsequently, a target image of a manipulable object appeared, and participants judged as quickly and accurately as possible whether the object afforded a precision- and power-grasping action by pressing the left or right key, respectively. The display terminated upon response or after 3 s.

Following each trial, an objective forced-choice discrimination screen assessed participants’ awareness of the cue stimulus. Using the up or down arrow keys, participants indicated whether the preceding cue had been “捏” (precision grasp) or “握” (power grasp) with the two options presented randomly above or below the center of the screen to prevent fixed stimulus–response mapping. The discrimination display was positioned away from the target-image region to avoid residual masking effects. To ensure precise reaction-time measurement, responses were collected using a dedicated directional response box, and participants used only their right hand. To avoid interference between action-judgment responses and awareness-test responses, distinct key mappings were employed for the two tasks. Participants were instructed to respond both quickly and accurately, regardless of confidence. Inter-trial intervals consisted of a blank black screen and varied randomly between 1.5 and 2 s.

The design included four within-participant conditions defined by semantic congruency (congruent/incongruent) and grasp type (precision grasp/power grasp). The experiment consisted of four blocks, each containing 128 trials. Half of the trials featured semantic congruence between the cue word and the grasping action afforded by the target object, and half were incongruent, with an equal distribution of precision- and power-grasps target stimuli across conditions.

### 2.4. EEG Data Acquisition

Continuous electroencephalogram (EEG) data were recorded using the Brain Vision Recorder 2.0 system (Brain Products, Gilching, Germany) with a 64-channel EasyCap arranged according to the international 10–20 system. The FCz electrode served as the online reference, and AFz was used as the ground. Vertical electrooculogram (VEOG) activity was recorded for subsequent removal of eye-movement artifacts. Signals were amplified using a BrainAmp amplifier (Brain Products, Gilching, Germany), digitized at 1000 Hz, and band-pass filtered from 0.01 to 100 Hz. Electrode impedances were kept below 5 kΩ throughout the recording session.

### 2.5. EEG Data Analysis

Offline EEG processing was conducted in EEGLAB (Delorme & Makeig, 2004; Iversen & Makeig, 2014) within MATLAB. During data acquisition, the FCz electrode served as the online reference. Prior to ERP analysis, all EEG data were re-referenced offline to the averaged signal of the left and right mastoid electrodes (TP9 and TP10). Independent component analysis (ICA) was applied to identify and remove ocular artifacts, following established procedures demonstrating its effectiveness for EOG correction (Gratton et al., 1983). Data were segmented from 200 ms before cue stimulus onset to 2000 ms after target stimulus onset. Trials containing major artifacts or voltage fluctuations exceeding ±80 μV were excluded. The data were low-pass filtered at 30 Hz, time-locked to target onset, and baseline-corrected to the 200 ms preceding cue presentation.

After preprocessing, ERP waveforms were averaged for each of the four experimental conditions defined by cue–target semantic congruency (congruent/incongruent) and grasp type (precision/power). The final number of valid trials included in each condition was 111 trials for congruent/precision, 112 for congruent/power, 110 for incongruent/precision, and 111 for incongruent/power.

Consistent with prior subliminal priming research, priming effects typically emerge as amplitude differences in occipital- and frontoparietal regions between 200 and 400 ms after target onset (Martens et al., 2011; Piotr et al., 2006; Zovko & Kiefer, 2013). Because the present study focused on semantic and action-related processing, three ERP components were examined: the P300, commonly associated with grasp-related stimulus evaluation (Lee et al., 2018); the N400, linked to semantic processing; and the P600, sensitive to semantic conflict. Accordingly, frontoparietal and parietal–occipital electrodes were selected as regions of interest.

Mean amplitudes were extracted from time-windows specific to each component: 280–330 ms for the P300 (CP1/CPz/CP2; P1/Pz/P2), 360–410 ms for the N400 (FC1/FCz/FC2; C1/Cz/C2), and 480–580 ms for the P600 (CP1/CPz/CP2; P1/Pz/P2). Repeated-measures ANOVAs were conducted on each component’s mean amplitude using SPSS 20.0 with Greenhouse–Geisser correction, including the factors region of interest, cue–target semantic congruency (congruent/incongruent), and grasping-action type (precision/power).

The time-windows used for ERP quantification were defined based on visual inspection of the grand-average ERP waveforms collapsed across all experimental conditions and participants. This procedure was adopted to avoid condition-specific bias in time-window selection. For the N400 component, a relatively narrow time-window was chosen, centered on the peak of the negative deflection observed at the centro-parietal electrodes, reflecting the temporal characteristics of the present data rather than a fixed canonical interval. The same time-windows were applied consistently across all conditions and participants in subsequent statistical analyses.

## 3. Results

### 3.1. Behavior

#### 3.1.1. An Objective Measure of Visual Awareness for Cue Stimuli

After each trial, participants reported their subjective awareness of the prime using the PAS. Only trials rated as PAS = 1 (no experience) were included in subsequent analyses. We further assessed visual awareness using a forced-choice discrimination test and Bayesian analysis in conjunction with conventional *p*-values. Analysis of their accuracy on the forced-choice screen, an objective discrimination test, revealed that their accuracy (mean ± SEM = 50.20 ± 0.02%) was at the chance level, approximately 50% (paired t test, t(29) = 1.602, *p* = 0.120, BF10 = 1.01), indicating no visual awareness of the cue stimulus. Consistent with this finding, signal-detection measures confirmed the absence of perceptual sensitivity. Further analysis of the discrimination index d′ and likelihood ratio beta revealed that d′ (mean ± SEM = 0.02 ± 0.003) did not significantly differ from 0 (paired t test, t(29) = 1.603, *p* = 0.120, BF10 = 0.60), and beta (mean ± SEM = 0.99 ± 0.001) did not differ from 1 (paired t test, t(29) = −1.283, *p* = 0.210, BF10 = 0.40). Taken together, these converging indicators demonstrate that participants were unable to discriminate or consciously perceive the masked priming stimuli, validating the subliminal nature of the cue words.

#### 3.1.2. Subliminal Priming Effect

Based on established priming theories, we expected that semantic congruence between the subliminal cue word and the grasping action depicted in the target would facilitate behavioral performance. In this experiment, we predicted that congruence between cue stimulus words and the grasping action of target objects would enhance the participants’ ability to judge these actions more effectively.

To ensure data quality, trials with a response time less than 200 ms or greater than 1500 ms were excluded, and data points exceeding two standard deviations from the mean were also removed. Additionally, trials in which participants made incorrect judgments were excluded from analysis. Only trials with correct responses regarding the object’s action type were included. Given the four experimental conditions and the repeated-measures design, the number of valid trials contributing to the final analyses was 121 trials for congruent/precision, 120 trials for congruent/power, 120 trials for incongruent/precision, and 120 trials for incongruent/power.

**Figure 3:**
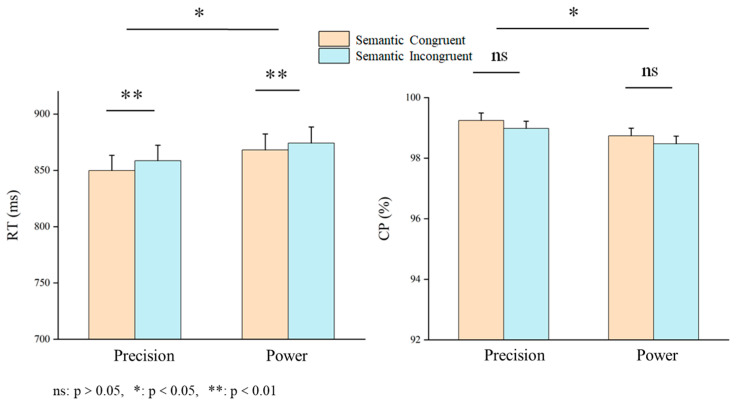
Results for the response time and accuracy. **Left**: ANOVA results for response time. The main effects of semantic consistency and action type are significant. **Right**: ANOVA results for accuracy. The main effect of action type is significant.

A 2 cue–target stimulus semantic congruency (congruent/incongruent) × 2 grasping action type (precision/power) repeated-measures ANOVA was used to examine object-recognition accuracy and response time. The ANOVA results for the response time indicated significant main effects for semantic consistency and action type. Specifically, the participants showed significantly faster reaction times (congruent: mean ± SEM = 858.97 ± 13.60 ms; incongruent: mean ± SEM = 866.41 ± 13.87 ms, F(1, 29) = 11.322, *p* = 0.002, η^2^*_p_* = 0.281, BF10 = 12.0) when the cue stimulus text matched the grasping action depicted in the target stimulus images than in the incongruent conditions (Figure 3). Also, the reaction time for precision is less than that for power (precision: mean ± SEM = 854.22 ± 13.57 ms; power: mean ± SEM = 871.16 ± 14.24 ms, F(1, 29) = 7.596, *p* = 0.010, η^2^*_p_* = 0.208, BF10 = 11.8).

Furthermore, the ANOVA results for accuracy showed a significant main effect of action type (precision: mean ± SEM = 98.99 ± 0.19%; power: mean ± SEM = 97.71 ± 0.19%, F(1, 29) = 7.361, *p* = 0.011, η^2^*_p_* = 0.202, BF10 = 11.8). However, there is no significant difference in interactions in response time (F(1, 29) = 0.292, *p* = 0.593, η^2^*_p_* = 0.010) and accuracy (F(1, 29) = 2.692, *p* = 0.112, η^2^*_p_* = 0.085). Also no significant semantic congruency effect in accuracy (F(1, 29) = 1.458, *p* = 0.237, η^2^*_p_* = 0.048).

### 3.2. Electrophysiology Components of the Subliminal Priming Task

**Figure 4:**
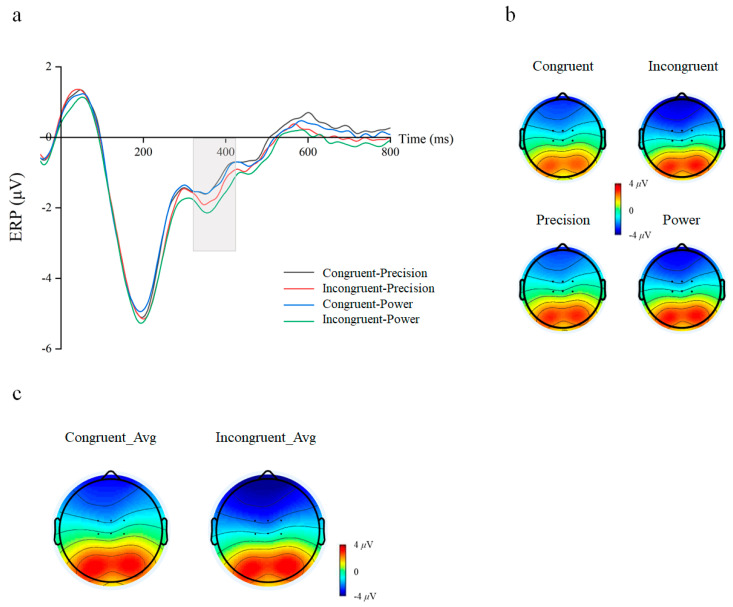
Waveforms and topographical maps for N400 component. (**a**) Average amplitude graphs for the four conditions at fronto-central electrodes (FC1/FCz/FC2/C1/Cz/C2). Gray rectangle refers to the window of interest. (**b**) Scalp topographies of the N400 activation in four conditions. (**c**) Scalp topographies of difference for congruency main effect.

A 2 cue–target stimulus congruency (congruent/incongruent) × 2 grasping action type (precision/power) repeated-measures ANOVA on the mean amplitude for the N400 component was performed. The result showed a significant main effect for Cue–Target Stimulus Congruency (congruent: mean ± SEM = −1.516 ± 0.19 μV; incongruent: mean ± SEM = −1.927 ± 0.18 μV, F(1, 29) = 29.726, *p* < 0.001, η^2^*_p_* = 0.506) on the mean amplitude. We also found that the action types’ main effect on the mean amplitude of N400 was significant. Under power-grasping action types, the amplitude of N400 activation was significantly greater than precision grasping action types (precision: mean ± SEM = −1.661 ± 0.18 μV; power: mean ± SEM = −1.782 ± 0.19 μV, F(1, 29) = 6.847, *p* = 0.014, η^2^*_p_* = 0.191) (Figure 4). There is no significant interaction (F(1, 29) = 2.484, *p* = 0.126, η^2^*_p_* = 0.079).

**Figure 5:**
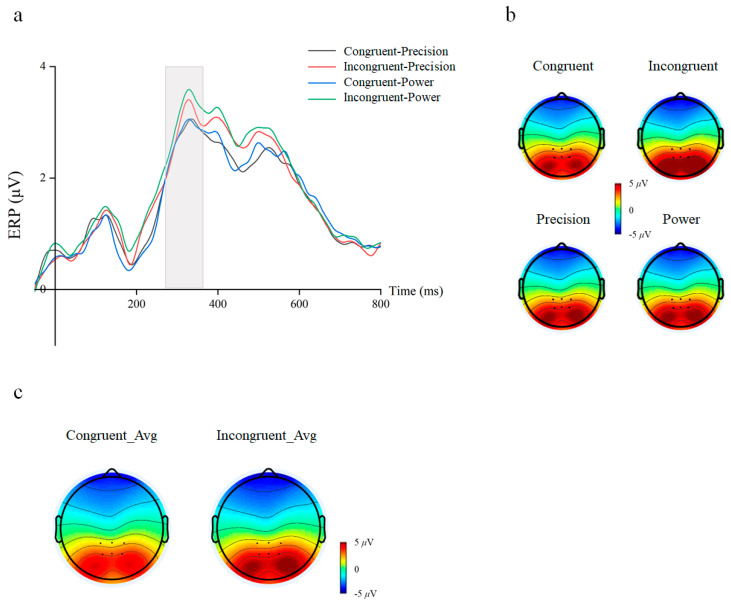
Waveforms and topographical maps for P300 component. (**a**) Average amplitude graphs for the four conditions at posterior parietal electrodes (CP1/CPz/CP2/P1/Pz/P2). Gray rectangle refers to the window of interest. (**b**) Scalp topographies of the P300 activation in four conditions. (**c**) Scalp topographies of difference for congruency main effect.

A 2 cue–target stimulus congruency (congruent/incongruent) × 2 grasping action type (precision/power) repeated-measures ANOVA on the mean amplitude for the P300 component was performed. The results revealed significant main effects for Cue–Target Stimulus Congruency (congruent: Mean ± SEM = 2.931 ± 0.31 μV, incongruent: Mean ± SEM = 3.305 ± 0.33 μV, F(1, 29) = 15.582, *p* < 0.001, η^2^*_p_* = 0.350) on the mean amplitude (Figure 5). However, the main effect of action types (F(1, 29) = 1.690, *p* = 0.204, η^2^*_p_* = 0.055) and interaction (F(1, 29) = 1.768, *p* = 0.194, η^2^*_p_* = 0.057) are not significant.

**Figure 6:**
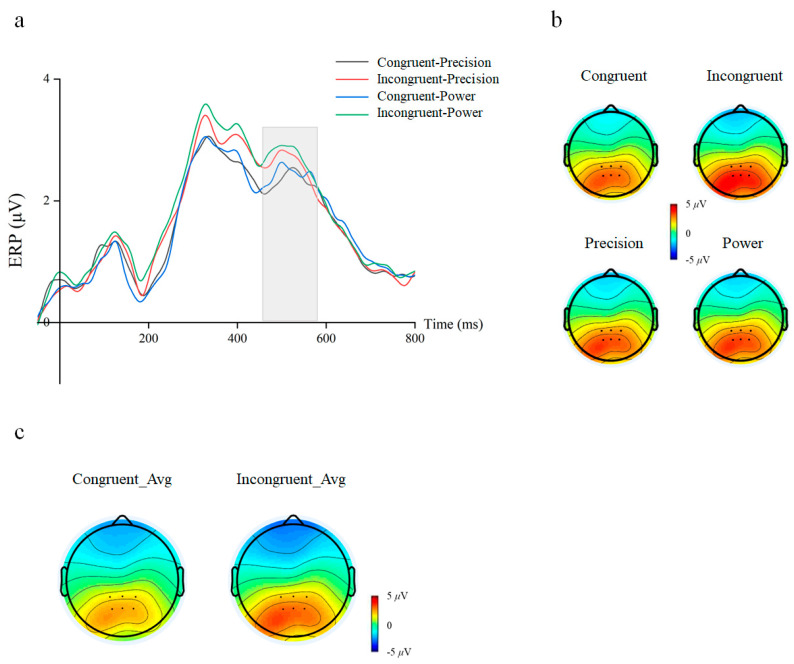
Waveforms and topographical maps for P600 component. (**a**) Average amplitude graphs for the four conditions at posterior parietal electrodes (CP1/CPz/CP2/P1/Pz/P2). Gray rectangle refers to the window of interest. (**b**) Scalp topographies of the P600 activation in four conditions. (**c**) Scalp topographies of difference for congruency main effect.

We performed 2 cue–target stimulus congruency (congruent/incongruent) × 2 grasping action types (precision/power) repeated-measures ANOVA on the mean amplitude for the P600 component, which revealed a significant main effect of semantic congruency (congruent: mean ± SEM = 2.470 ± 0.22 μV; incongruent: mean ± SEM = 2.834 ± 0.24 μV, F(1, 29) = 8.265, *p* = 0.007, η^2^*_p_* = 0.222) (Figure 6). However, the main effect of action types (F(1, 29) = 2.547, *p* = 0.121, η^2^*_p_* = 0.081) and interaction (F(1, 29) = 0.022, *p* = 0.875, η^2^*_p_* = 0.001) are not significant.

## 4. Discussion

This study examined whether grasping-related action verbs undergo semantic processing without visual awareness. Using masked priming, we suppressed conscious access to verbal cues and assessed their influence on the recognition of grasping actions. Behaviorally, semantically congruent cue–target pairs produced faster grasp-type judgments, demonstrating a robust subliminal priming effect. ERP analyses further identified congruency-related modulation in components associated with semantic processing (N400), conflict monitoring (P600), and action-related evaluative processes (P300). Together, these findings provide convergent evidence that the semantic content of grasp-related verbs is activated automatically and can guide subsequent recognition of object affordances.

### 4.1. Subthreshold Semantic Priming Effect of Action Verbs

The N400 component, a hallmark of lexical–semantic processing (Brown & Hagoort, 1993; Dudschig, 2022; Kutas & Federmeier, 2011), showed greater amplitudes for incongruent cue–target pairs, indicating increased semantic integration difficulty. This aligns with subliminal priming literature (Kiefer & Brendel, 2006; Yang et al., 2017) and supports spreading activation accounts (Collins et al., 1975; Ortu et al., 2013), which predict facilitated processing for conceptually related stimuli (Federico et al., 2021). The effectiveness of action–semantic stimulation is further consistent with recent visual exploration findings and action–semantic ERP studies (Ball et al., 2025; Leynes et al., 2024).

The P600 component, commonly linked to semantic conflict and reanalysis (Emmorey et al., 2025; Kim et al., 2024), was also amplified for incongruent pairings. This pattern suggests that subliminal verb cues produce detectable semantic inconsistencies that require additional psychological resources for resolution, consistent with slower behavioral responses under incongruent conditions.

In addition, the P300 component—associated with action classification, stimulus evaluation, and decision-making (Danielle et al., 2019)—was sensitive to cue–target congruency. Larger P300 amplitudes for incongruent conditions reflect increased cognitive demands and align with prior findings that grasp-feature stimuli evoke P300 modulation (Jena et al., 2022; Lee et al., 2018). These convergent ERP effects demonstrate that grasping-action semantics are automatically activated and influence perceptual–motor evaluations, even when presented below the threshold of awareness. These findings highlight the association of manipulation-action cognition with the visual cortex and occipito–temporal lobe (Garcea & Buxbaum, 2019).

Integrating the behavioral outcomes with the ERP amplitude patterns, our findings indicate that the semantic congruency of subliminal grasp-related verbs produces a reliable facilitatory priming effect on action-type identification. This observation is consistent with established conclusions from semantic priming paradigms (Plaut & Erlbaums, 1998) and with the action–sentence compatibility effects frequently reported in embodied cognition research (Dam, 2010). Importantly, even though the present study employed single action verbs rather than full sentences—thereby simplifying the semantic structure—the priming effect remained robust. This further supports the view that action-related verbs inherently carry embodied semantic properties (Courson & Tremblay, 2020; Giacobbe et al., 2022).

The ERP results corroborate and extend these behavioral findings. We observed clear P300, N400, and P600 components in the predefined regions of interest, and their amplitudes were systematically modulated by the semantic congruency of the grasp-related cues. This pattern suggests that subliminal semantic conflict triggers increased neural engagement, a conclusion that aligns with prior work demonstrating that incongruent semantic information requires additional cognitive resources and consequently elicits larger neural responses (Federico et al., 2021). From a cognitive–neuroscientific perspective, our results also lend support to theoretical models proposing that grasp-action processing follows a functional pathway extending from occipital visual areas through the parietal cortex to frontal motor regions—particularly involving the dorso–dorsal stream of the dorsal pathway (Augurelle et al., 2003). Furthermore, the modulation of later occipitoparietal activity by semantic congruency suggests that higher-order cognitive operations remain sensitive to semantic conflict even when the initiating cue is presented below conscious awareness.

It should be noted that the use of ERP methodology in the present study does not permit precise spatial localization of the neural generators underlying the observed effects. Accordingly, any references to dorsal- or ventral processing streams should be interpreted at a conceptual level rather than as direct neuroanatomical evidence. Future studies employing methods with higher spatial resolution, such as fMRI or combined EEG–fMRI approaches, will be necessary to more precisely characterize the neural pathways involved in subliminal processing of grasp-related language.

Within this constraint, it is important to distinguish between action-control mechanisms and semantic representations. The dorso–dorsal stream has been proposed to support online visuomotor control and the de novo generation of grasping actions, without necessarily relying on long-term semantic knowledge of specific grasp-types. In contrast, semantic information is typically associated with ventral-stream processing, which supports object-related and conceptual representations.

From the perspective of integrative models such as the Three-Action System framework, action-related and semantic systems are partially dissociable yet dynamically interacting (Federico et al., 2023, 2025; Seidel et al., 2023). Accordingly, the present findings cannot be taken as evidence that semantic processing alone drives the observed effects. Instead, they suggest that during grasp-type judgments—particularly for familiar objects—performance may rely on a combination of action-related processing and semantic information, even under subliminal conditions. The relative contribution of these systems remains an open question for future research.

### 4.2. Action Language and Action Recognition

In addition to the robust effect of semantic congruency, a main effect of grasp type was observed across behavioral measures and N400 amplitudes, indicating that different categories of grasping actions are associated with distinct processing characteristics. Importantly, because the interaction between semantic congruency and grasp type was not statistically significant, this effect should not be interpreted as evidence for differential semantic priming strength across grasp types.

Instead, the grasp-type effect is best viewed as a complementary finding, suggesting that the cognitive system is sensitive to categorical differences in action representations during grasp judgment (Bergstrom et al., 2021). This effect does not add decisive support to any specific theory of semantic representation but is compatible with integrative accounts in which semantic- and action-related information are jointly processed. In contrast, the consistent differences between congruent and incongruent conditions across grasp types constitute the primary evidence for subliminal semantic processing of action-related language.

These grasp-type differences contribute important nuance to embodied cognition frameworks. Prior accounts suggest that action execution and action-language processing rely on partially overlapping neural systems (Courson & Tremblay, 2020; Giacobbe et al., 2022). The present findings extend this view by demonstrating that motoric distinctions between grasp types manifest even when semantic processing occurs outside conscious awareness. In line with this perspective, our results provide further evidence that subliminal action semantics still engage motor-related cognitive mechanisms.

From a theoretical perspective, the present findings do not allow strong claims regarding whether action-related meanings are grounded in sensorimotor representations or instantiated in an abstract semantic system. Rather, our results are best interpreted within integrative accounts that emphasize interactions between partially dissociable systems. One such framework is the Three-Action System (3AS) model, which proposes that action representation relies on both dorsal-stream action systems and ventral-stream semantic systems that can operate independently but interact dynamically during action understanding and recognition (Federico et al., 2023, 2025; Seidel et al., 2023).

Within this framework, linguistic information about actions can influence action recognition without requiring a strong form of sensorimotor grounding. Empirical evidence supports this view, showing that even when nouns are used as stimuli, words referring to highly manipulable objects can enhance memory performance, reflecting functional associations between semantic- and action-related representations (Klepp et al., 2014). The present findings extend this line of work by demonstrating that grasp-related verbs can exert semantic effects under subliminal conditions and that different grasp types (precision vs. power grasping) are differentially processed.

Importantly, these effects do not imply that action meaning is necessarily grounded in sensorimotor representations. Instead, they suggest that semantic- and action-related information can be integrated during early processing stages, even in the absence of conscious awareness. This interpretation is compatible with embodied, amodal, and hybrid theories of semantic representation, and future studies combining linguistic priming with direct sensorimotor manipulations will be necessary to further clarify the representational architecture of action semantics (Wu et al., 2013).

Moreover, the observed grasp-type asymmetries map closely onto principles of First-order planning highlighted in the action cognition literature. Smaller objects generally afford a finger-closing movement characteristic of precision actions, whereas larger objects typically require an opening of the hand consistent with power-grasp actions. Variations in the motoric complexity or biomechanical demands of these grasp types likely contribute to differences in recognition performance, including the reaction time advantages observed for precision actions (Brandi et al., 2014; Rosenbaum et al., 2012). These findings thus suggest that even during rapid- and subliminal processing, the cognitive system flexibly integrates low-level motor preferences with high-level semantic representations.

However, the current study employed a relatively limited action taxonomy. Although semantic differences emerged in the ERP data, it remains unclear whether these differences stem solely from grasp-type semantics or from linguistic properties of the verbs themselves. Future studies should employ more fine-grained classifications of grasp actions—including biomechanical complexity, force requirements, and digit patterns—to better distinguish motoric from lexical influences. Expanding beyond finger-configurational grasp types will clarify how deeply subliminal action-language processing mirrors motor-action structure.

Overall, by using Chinese action verbs instead of visual action depictions, the present study provides compelling evidence for subliminal semantic activation of grasping actions from a language-based perspective.

## 5. Conclusions

This study demonstrates that grasping-action semantics can be processed unconsciously under masked verbal priming. Congruent cue–target pairs facilitated behavioral performance, while incongruent pairs elicited larger N400, P300, and P600 amplitudes, reflecting greater semantic and cognitive demands. Differences between precision- and power-grasping actions further showed that grasp types impose distinct processing requirements even below awareness.

Collectively, these findings support embodied accounts of action-semantic processing. The results offer a clear foundation for future work examining the mechanisms and constraints of subliminal action semantics and the neural pathways supporting unconscious understanding of manipulative actions.

## Figures and Tables

**Table 1:** Chi-square test for the choice of grasping-action types for eight objects.

Manipulable Objects	Grasping-Action Types (Sample Number = 198)		Chi-Square Test (Chi-Square Value, *p* Value)
	Precision	Power	Grasping-Action Types
Brush	195	3	186.18, <0.001
Fan	193	5	178.92, <0.001
Hammer	0	198	198, <0.001
Dryer	2	196	190.94, <0.001
Clamp	196	2	190.94, <0.001
Scissor	198	0	198, <0.001
Stopwatch	2	196	190.94, <0.001
Bottle	3	195	186.18, <0.001

## Data Availability

The data from this study have been reflected in the text.
